# Supplementary material for: Internalization and Transport of PEGylated Lipid-Based Mixed Micelles across Caco-2 Cells Mediated by Scavenger Receptor B1
Source: Pharmaceutics. 2021 Nov 26;13(12):2022. doi: 10.3390/pharmaceutics13122022 (PMC8703698; doi:10.3390/pharmaceutics13122022)
Supplement: Supplementary file 1 [file pharmaceutics-13-02022-s001.zip › pharmaceutics-1452617-supplementary for conversion.pdf]

pharmaceutics

MDPI

Supplementary Materials

Internalization and Transport of PEGylated Lipid-Based Mixed Micelles Across Caco-2 Cells Mediated by Scavenger Receptor B1

Xiangjie Su [1], Mercedes Ramírez-Escudero [2], Feilong Sun [1], Joep B. van den Dikkenberg [1], Mies J. van Steenbergen [1], Roland J. Pieters [3], Bert J. C. Janssen [2], Peter M. van Hasselt [4], Wim E. Hennink [1] and Cornelus F. van Nostrum [1,*]

[1] Department of Pharmaceutics, Utrecht Institute for Pharmaceutical Sciences, Utrecht University, 3584 CG Utrecht, The Netherlands; x.su1@uu.nl (X.S); dragonfly88214@hotmail.com (F.S.); j.b.vandendikkenberg@uu.nl (J.B.v.d.D.); m.j.vansteenbergen@uu.nl (M.J.v.S.); w.e.hennink@uu.nl (W.E.H.)

[2] Structural Biochemistry, Bijvoet Center for Biomolecular Research, Faculty of Science, Utrecht University, 3584 CG Utrecht, The Netherlands. mramirezescudero@gmail.com (M.R.E.); b.j.c.janssen@uu.nl (B.J.C.J.)

[3] Department of Chemical Biology & Drug Discovery, Utrecht Institute for Pharmaceutical Sciences, Utrecht University, 3584 CG Utrecht, The Netherlands; R.J.Pieters@uu.nl

[4] Department of Pediatrics, Wilhelmina Children's Hospital, University Medical Center Utrecht, 3584 EA Utrecht, The Netherlands; P.vanhasselt@umcutrecht.nl

* Correspondence: C.F.vanNostrum@uu.nl; Tel.: +31-620274607

Z-average size

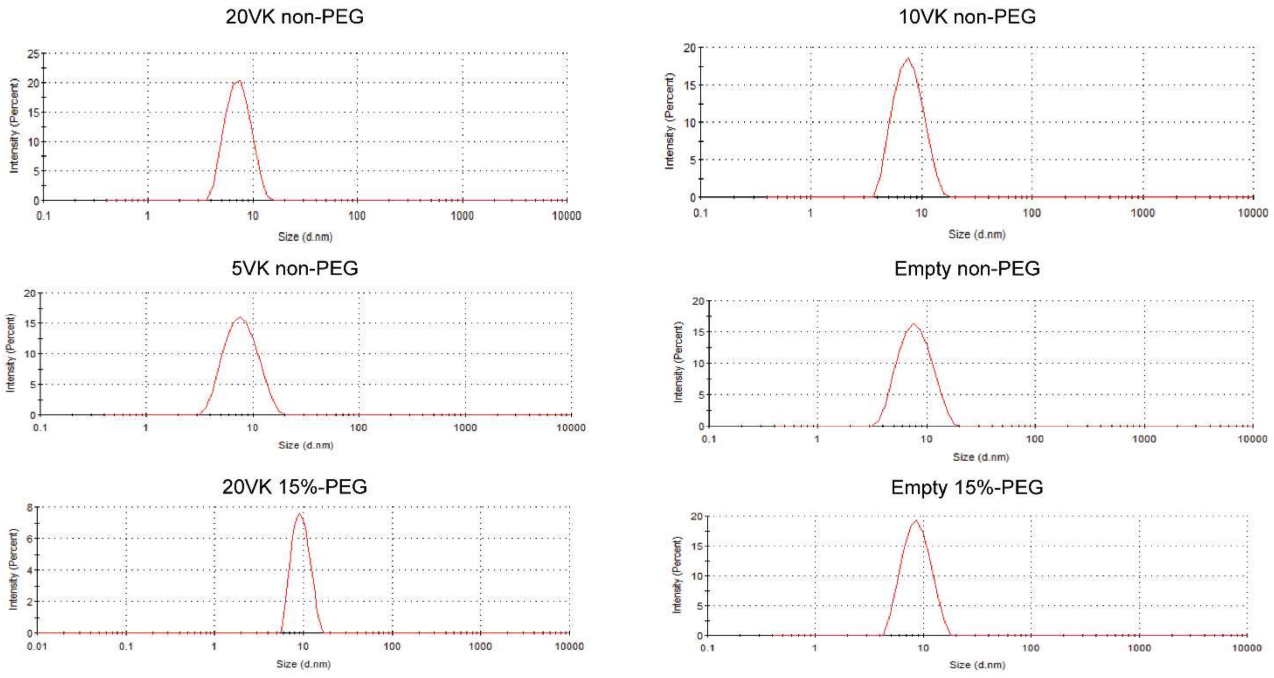

20VK 30%-PEG

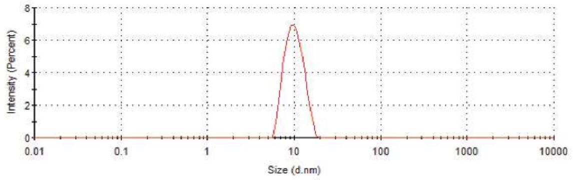

Blank 30%-PEG

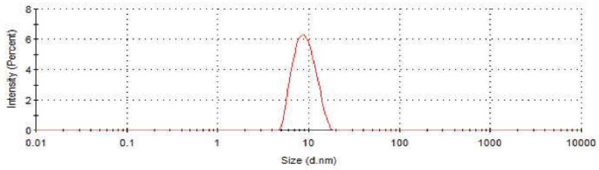

20VK 50%-PEG

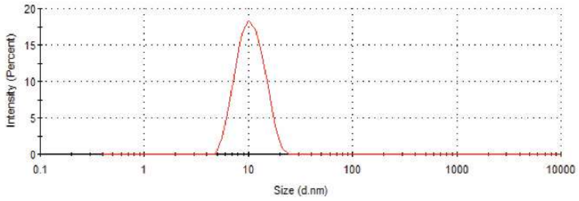

Empty 50%-PEG

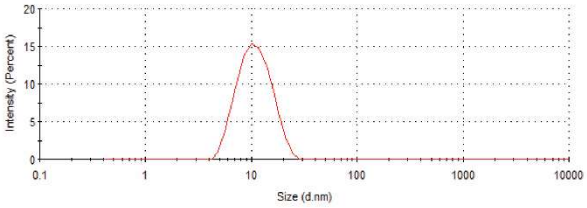

DSPE-PEG

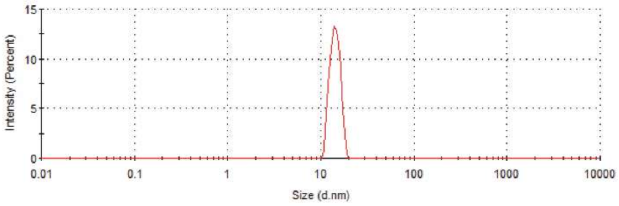

Zeta potential

20VK non-PEG

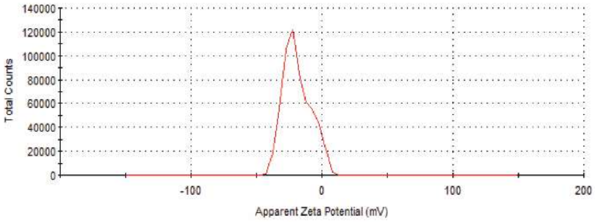

10VK non-PEG

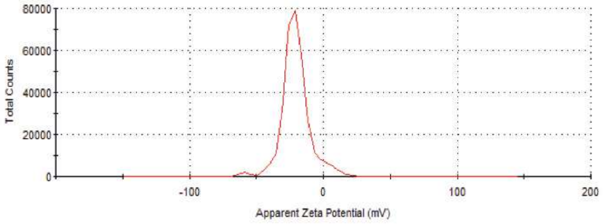

5VK non-PEG

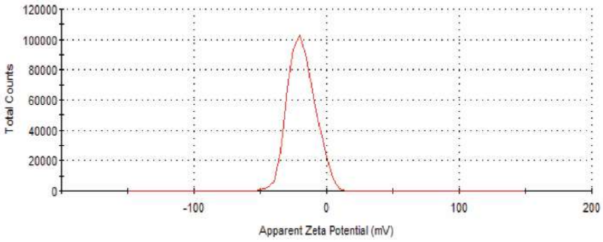

Epmty non-PEG

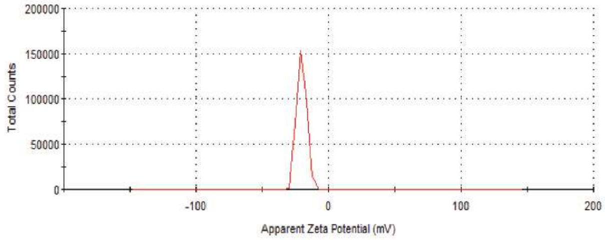

20VK 15%-PEG

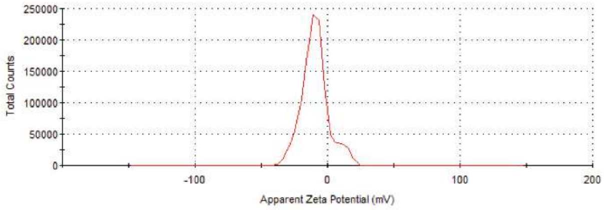

Epmty 15%-PEG

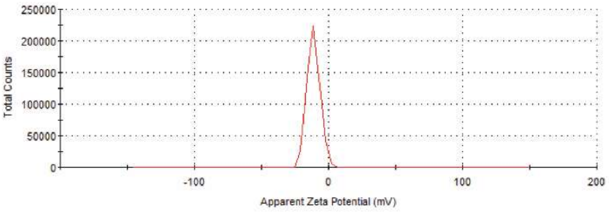

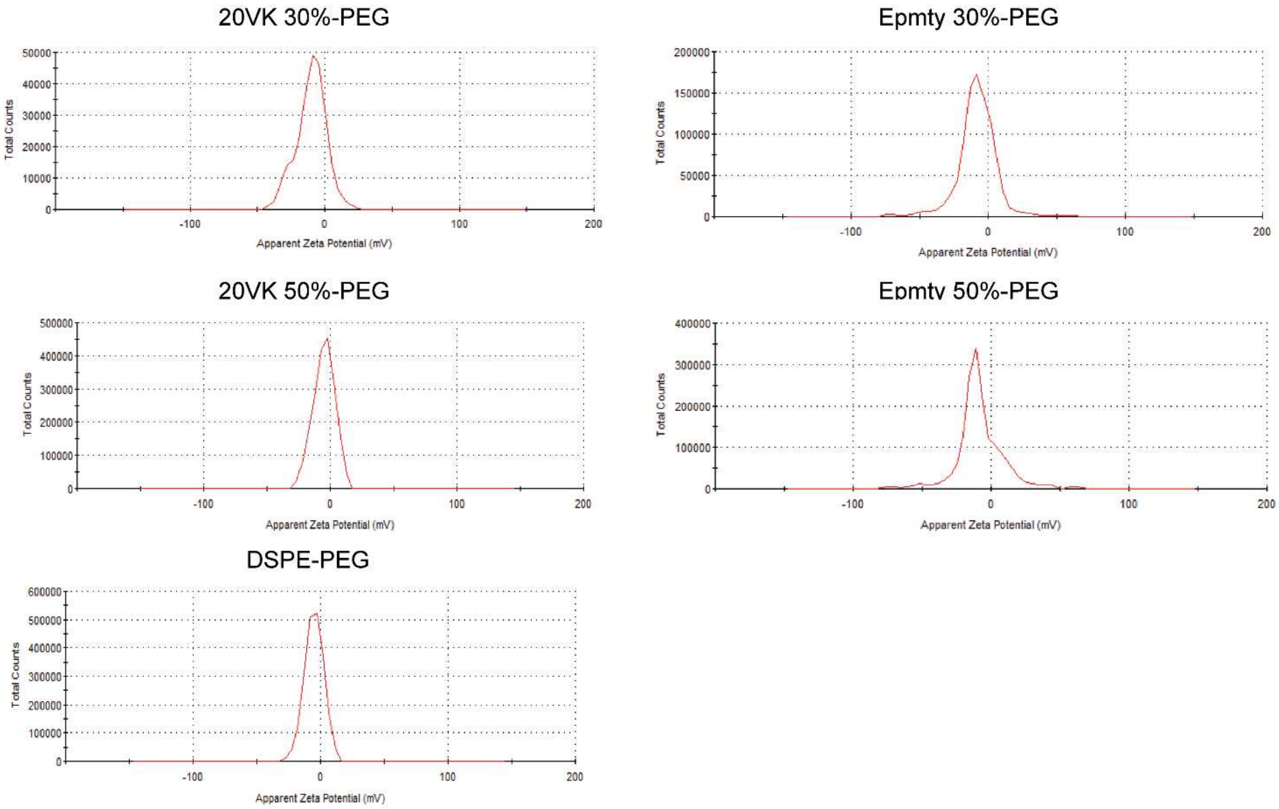

Figure S1. Representative Z-average size and zeta potential of mixed micelles with varying vitamin K loading and PEG content

Table S1. Calculated distance between neighboring chains (*D*) and *Rf*/*D* of micellar PEG layer

Formulations	Radius of Micelle* (nm)	wt% PEG	*D*(nm)	R_f/D
20VK-15%-PEG	4.55	17.5%	2.61	1.32
Empty 15%-PEG	4.20	18.5%	2.86	1.20
20VK-30%-PEG	4.85	29.7%	1.94	1.78
Empty 30%-PEG	4.35	31.2%	2.09	1.65
20VK-50%-PEG	5.35	41.2%	1.33	2.58
Empty 50%-PEG	5.25	42.9%	1.34	2.56
DSPE-PEG	7.05	71.3%	0.67	5.13

* Radius of micelle as determined by DLS, Rf = 3.44.

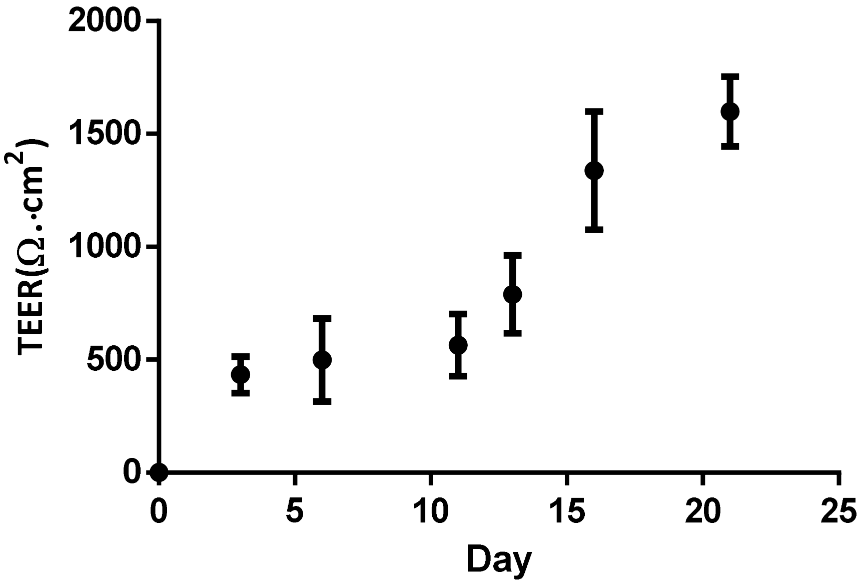

Figure S2. Trans-epithelial electric resistance (TEER) values (mean ± SD from 24 wells) of Caco-2 monolayers on transwell inserts as a function of the culture time.

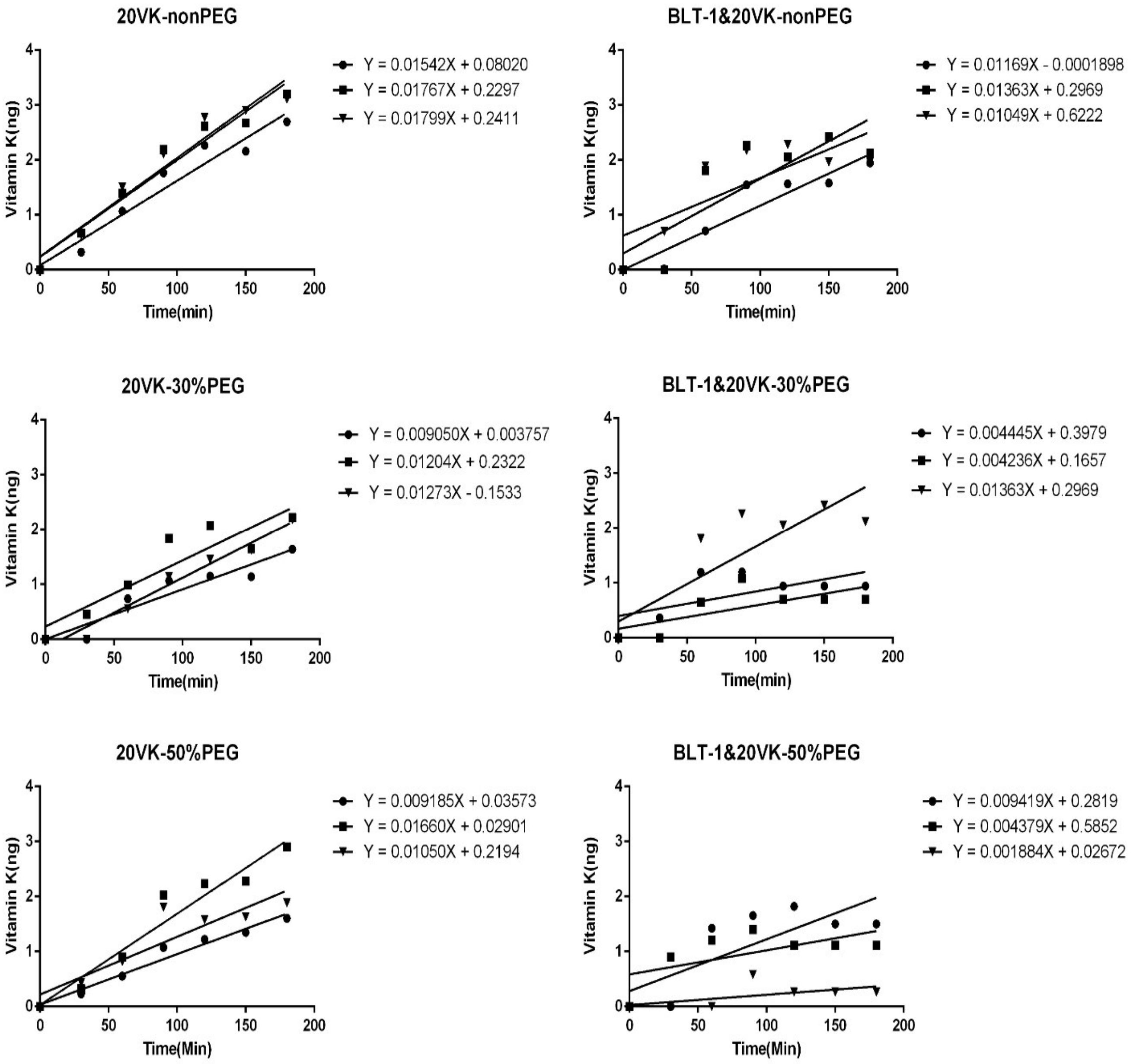

Figure S3. Cumulative amounts of transported vitamin K across Caco-2 monolayers. From top to bottom: non-PEGylated micelles, 30%-PEGylated micelles and 50%-PEGylated micelles; without (left) and with 10 μM BLT-1 inhibitor (right) (triplicates for each group).

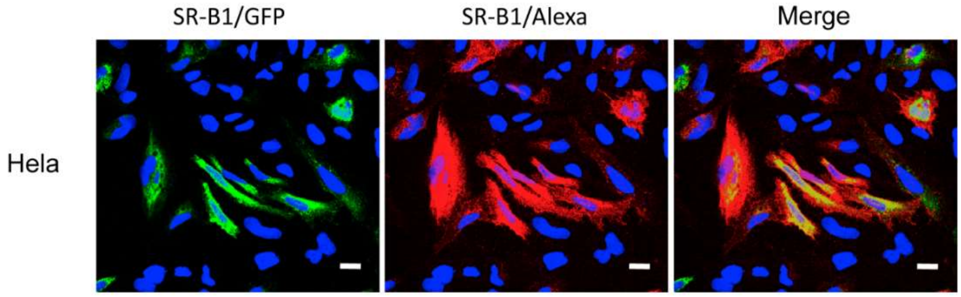

Figure S4. Fluorescence of the SR-B1/GFP fusion protein and immunofluorescence staining of SR-B1/Alexa in transfected HELA cells (the scale bar is 20 μm).

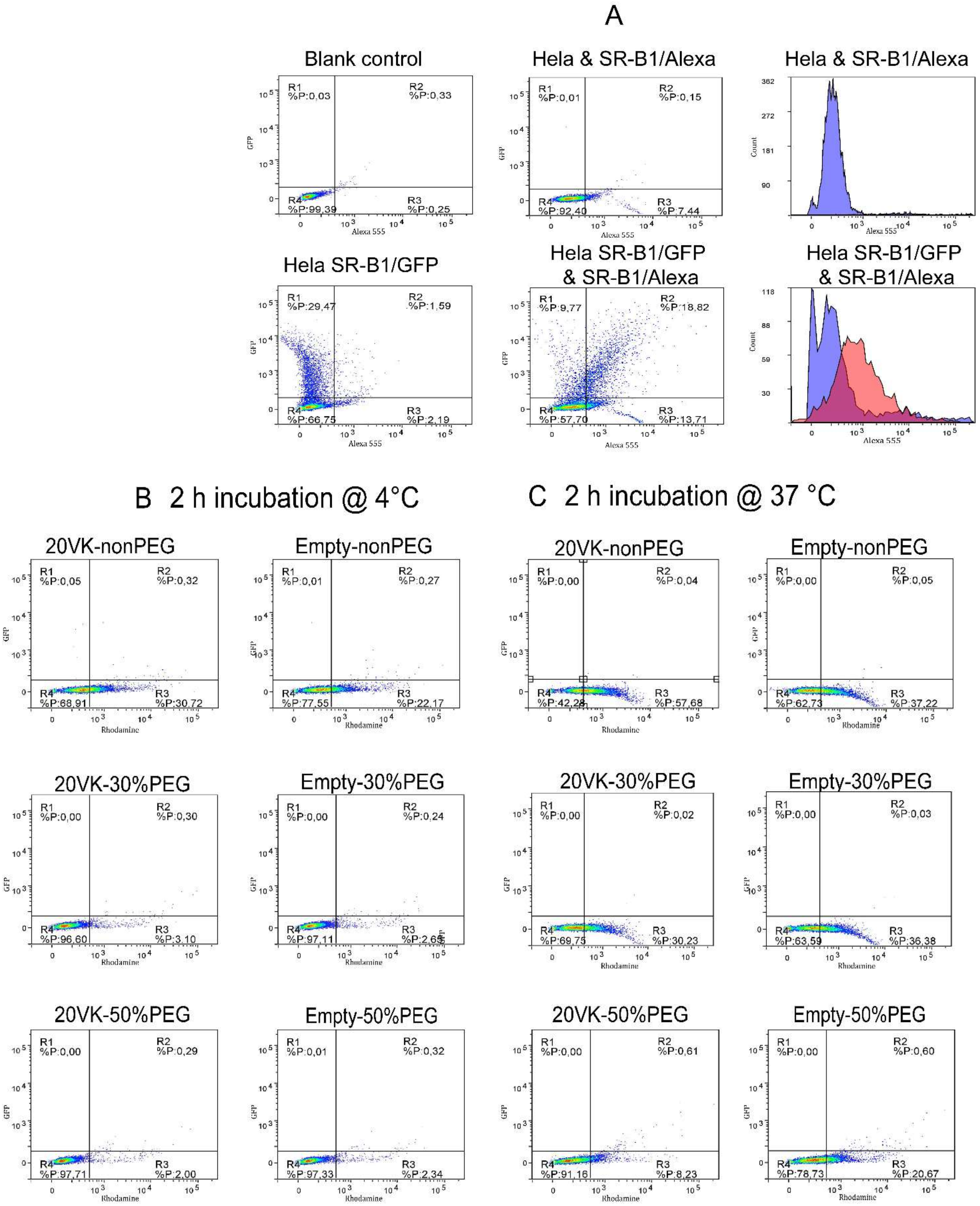

A
Blank control
R1 %P:0,03
R2 %P:0,33
R4 %P:99,39
R3 %P:0,25
Hela & SR-B1/Alexa
R1 %P:0,01
R2 %P:0,15
R4 %P:92,40
R3 %P:7,44
Hela & SR-B1/Alexa
Hela SR-B1/GFP
R1 %P:29,47
R2 %P:1,59
R4 %P:66,75
R3 %P:2,19
Hela SR-B1/GFP & SR-B1/Alexa
R1 %P:9,77
R2 %P:18,82
R4 %P:57,70
R3 %P:13,71
Hela SR-B1/GFP & SR-B1/Alexa
GFP
Alexa 555
Count
B 2 h incubation @ 4°C
C 2 h incubation @ 37 °C
20VK-nonPEG
R1 %P:0,05
R2 %P:0,32
R4 %P:68,91
R3 %P:30,72
Empty-nonPEG
R1 %P:0,01
R2 %P:0,27
R4 %P:77,55
R3 %P:22,17
20VK-nonPEG
R1 %P:0,00
R2 %P:0,04
R4 %P:42,28
R3 %P:57,68
Empty-nonPEG
R1 %P:0,00
R2 %P:0,05
R4 %P:62,73
R3 %P:37,22
20VK-30%PEG
R1 %P:0,00
R2 %P:0,30
R4 %P:96,60
R3 %P:3,10
Empty-30%PEG
R1 %P:0,00
R2 %P:0,24
R4 %P:97,11
R3 %P:2,65
20VK-30%PEG
R1 %P:0,00
R2 %P:0,02
R4 %P:69,75
R3 %P:30,23
Empty-30%PEG
R1 %P:0,00
R2 %P:0,03
R4 %P:63,59
R3 %P:36,38
20VK-50%PEG
R1 %P:0,00
R2 %P:0,29
R4 %P:97,71
R3 %P:2,00
Empty-50%PEG
R1 %P:0,01
R2 %P:0,32
R4 %P:97,33
R3 %P:2,34
20VK-50%PEG
R1 %P:0,00
R2 %P:0,61
R4 %P:91,16
R3 %P:8,23
Empty-50%PEG
R1 %P:0,00
R2 %P:0,60
R4 %P:78,73
R3 %P:20,67
Rhodamine

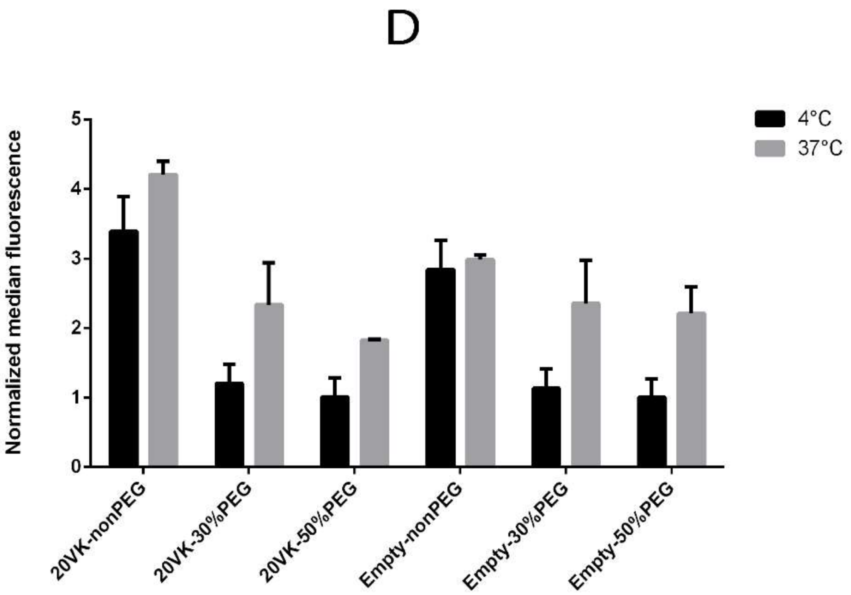

Figure S5. (**A**) FACS analysis of Hela cells showing SR-B1/GFP fluorescence (Y-axis) and/or SR-B1 expression as determined by immunofluorescence staining (X-axis) (Top: native Hela cells, Bottom: transfected Hela cells). GFP/SR-B1 fluorescence(Y-axis) and rhodamine fluorescence (X-axis) upon incubation at 4 °C (**B**) and at 37 °C (**C**) with native Hela cells of, from top to bottom: non-PEGylated, 30%-PEGylated and 50%-PEGylated mixed micelles (1.58 mM lipid) labeled with rhodamine without (empty) or loaded with vitamin K (350 μM). (**D**) Quantification of corresponding fluorescence of rhodamine for native HeLa cells incubated with micellar formulations, shown as mean ± SD. The quantified fluorescence intensity was normalized by the intensity observed for cells incubated with 20VK-50%PEG micelles.

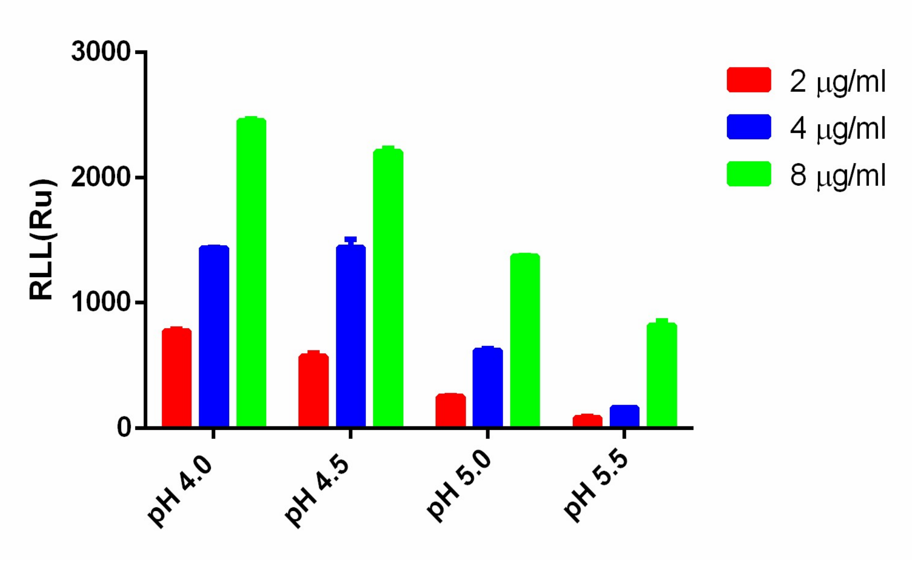

Figure S6. RLL (response of local ligand) values of SR-B1 spots immobilized on the chip using different SR-B1 solutions in PBS (2, 4 and 8 μg/mL) at four different pHs (4.0, 4.5, 5.0, 5.5).

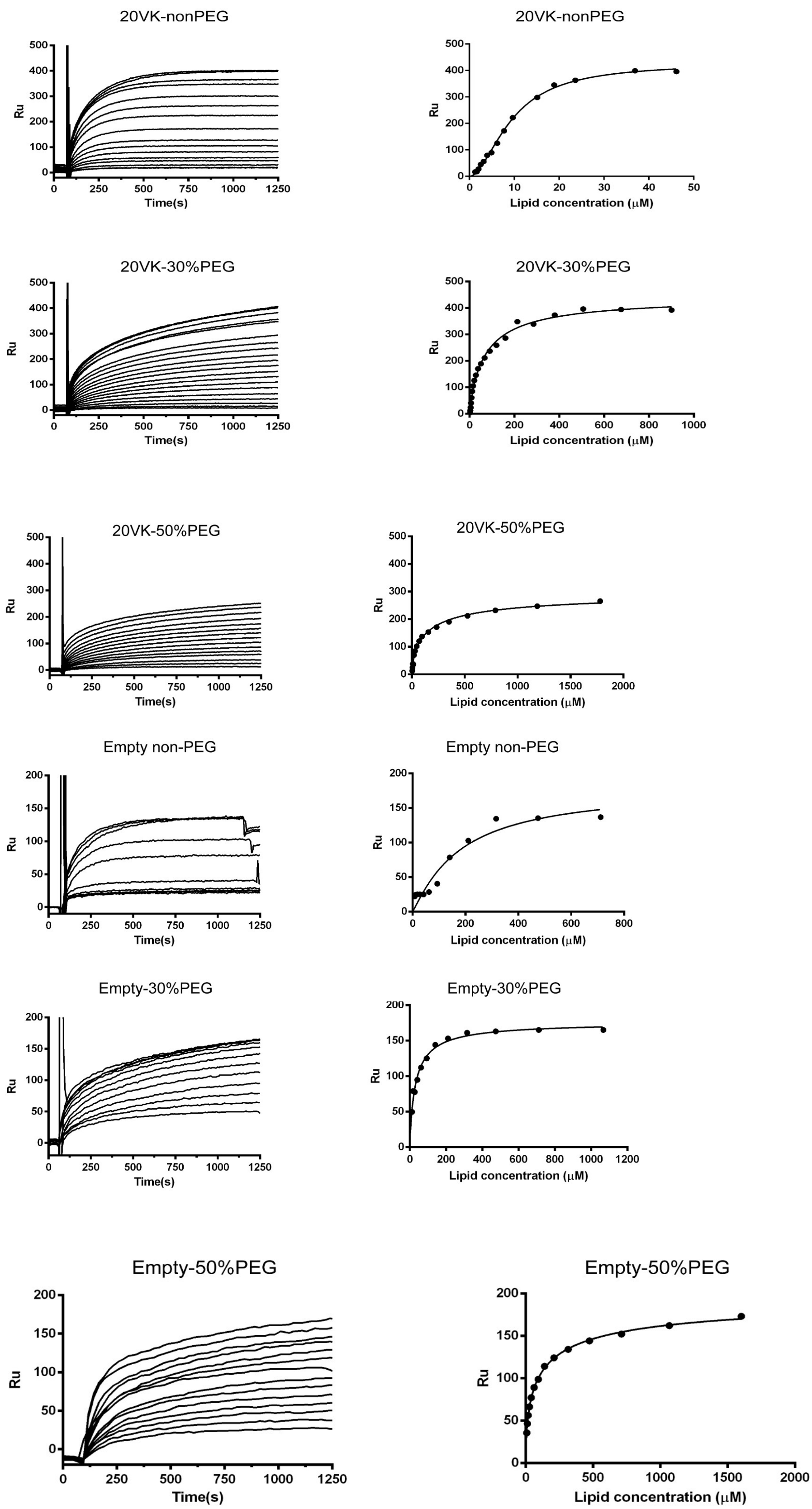

Figure S7. The representative binding responses of serial dilutions of mixed micelles (non-PEG, 30%-PEG and 50%-PEG without (empty) and with 20 mg vitamin K) interacting with SR-B1immobilized on the SPR chip (left) and fitting of SPR data to one site specific model (right). The response was recorded at spots with RLL 2452 ± 16.

A

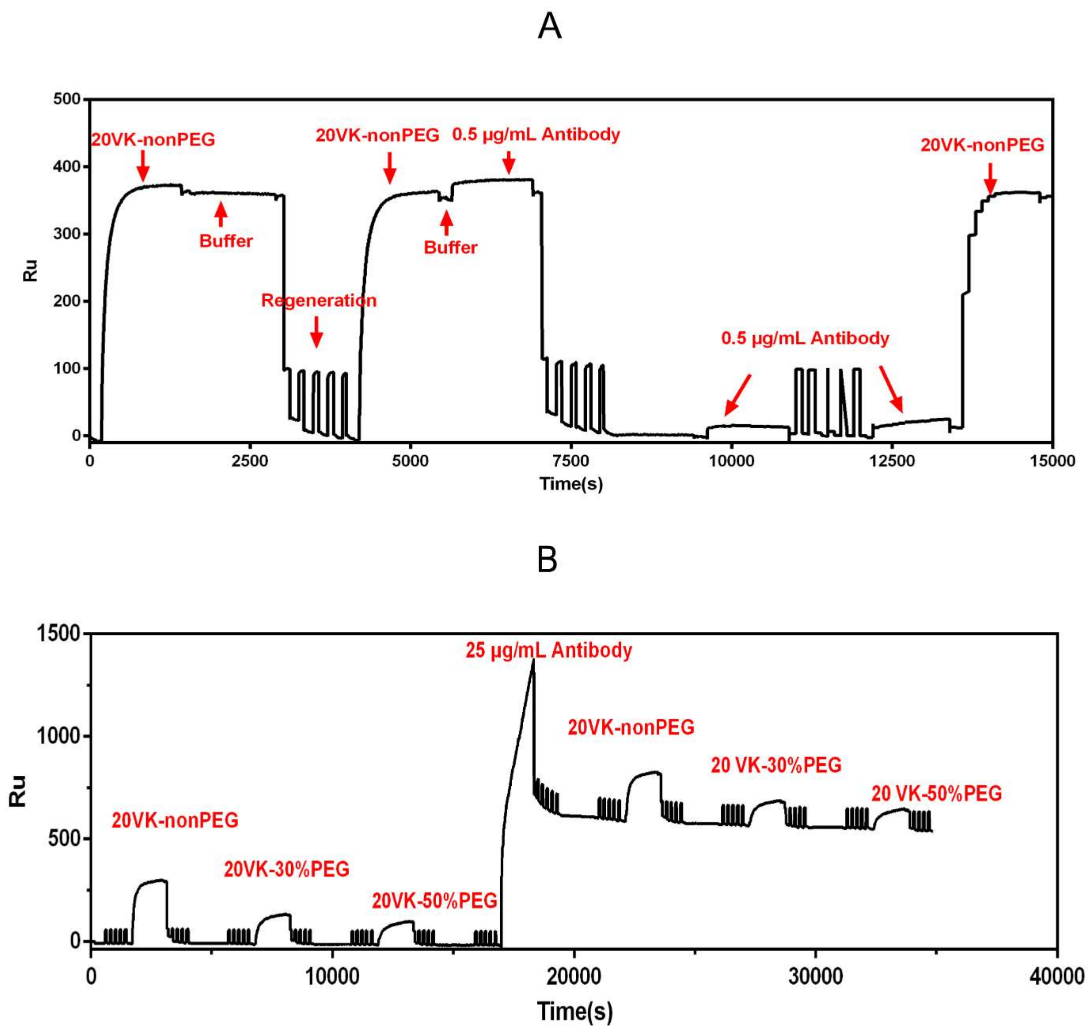

Figure S8. Competition between vitamin K loaded mixed micelles and a matching antibody for their interaction with the SR-B1. (**A**) Subsequent injections of 20VK-nonPEG formulations (8 µM vitamin K, 36 µM lipid) and 0.5 µg/mL antibody: antibody was first exposed to the occupied receptor (after second injection of the micelles), then twice to the regenerated receptor, after which the micelles were injected again. (**B**) SPR response of micelles with different degrees of PEGylation (20VK-nonPEG, 20VK-30PEG, 20VK-50PEG, with 8 µM vitamin K and 36 µM lipid) before and after the SR-B1 was blocked by 25µg/mL antibody.
